# Ellagitannins from *Rubus* Berries for the Control of Gastric Inflammation: *In Vitro* and *In Vivo* Studies

**DOI:** 10.1371/journal.pone.0071762

**Published:** 2013-08-05

**Authors:** Enrico Sangiovanni, Urska Vrhovsek, Giuseppe Rossoni, Elisa Colombo, Cecilia Brunelli, Laura Brembati, Silvio Trivulzio, Mattia Gasperotti, Fulvio Mattivi, Enrica Bosisio, Mario Dell'Agli

**Affiliations:** 1 Department of Pharmacological and Biomolecular Sciences, Università degli Studi di Milano, Via Balzaretti, Milano, Italy; 2 Department of Medical Biotechnology and Translational Medicine, Università degli Studi di Milano, Milano, Italy; 3 Department of Food Quality and Nutrition, Research and Innovation Centre, Fondazione Edmund Mach (FEM), S. Michele all'Adige (TN), Italy; Virginia Tech, United States of America

## Abstract

Ellagitannins have shown anti-inflammatory and anti-*Helicobacter pylori* properties; however, their anti-inflammatory activity at gastric level was not previously investigated. The aim of this research was to evaluate the effects of ellagitannins from *Rubus* berries on gastric inflammation. Ellagitannin enriched extracts (ETs) were prepared from *Rubus fruticosus* L. (blackberry) and *Rubus idaeus* L. (raspberry). The anti-inflammatory activity was tested on gastric cell line AGS stimulated by TNF-α and IL-1β for evaluating the effect on NF-kB driven transcription, nuclear translocation and IL-8 secretion. *In vivo* the protective effect of ellagitannins was evaluated in a rat model of ethanol-induced gastric lesions. Rats were treated orally for ten days with 20 mg/kg/day of ETs, and ethanol was given one hour before the sacrifice. Gastric mucosa was isolated and used for the determination of IL-8 release, NF-kB nuclear translocation, Trolox equivalents, superoxide dismutase and catalase activities. *In vitro*, ETs inhibited TNF-α induced NF-kB driven transcription (IC_50_: 0.67–1.73 µg/mL) and reduced TNF-α-induced NF-kB nuclear translocation (57%–67% at 2 µg/mL). ETs inhibited IL-8 secretion induced by TNF-α and IL-1β at low concentrations (IC_50_ range of 0.7–4 µg/mL). Sanguiin H-6 and lambertianin C, the major ETs present in the extracts, were found to be responsible, at least in part, for the effect of the mixtures. ETs of blackberry and raspberry decreased Ulcer Index by 88% and 75% respectively and protected from the ethanol induced oxidative stress in rats. CINC-1 (the rat homologue of IL-8) secretion in the gastric mucosa was reduced in the animals receiving blackberry and raspberry ETs. The effect of ETs on CINC-1 was associated to a decrease of NF-κB nuclear translocation in ETs treated animals. The results of the present study report for the first time the preventing effect of ETs in gastric inflammation and support for their use in dietary regimens against peptic ulcer.

## Introduction

The gastrointestinal tract represents an important barrier between the human hosts and microbial populations. One potential consequence of host-microbial interactions is the development of mucosal inflammation, which can lead to gastritis and ulcer. *Helicobacter pylori* (*H. pylori*), a Gram-negative pathogen that colonizes the stomach of humans and primates, is the most responsible for these inflammatory processes. *H. pylori* infection in humans represents a serious public health concern: the WHO classifies this bacterium as a Type 1 carcinogen. The clinical course of *H. pylori* infection is highly variable and is influenced by both microbial and host factors. The pattern and distribution of gastritis strongly correlate with the risk of clinical duodenal or gastric ulcers, mucosal atrophy, gastric carcinoma, or gastric lymphoma.

It has been demonstrated that gastric epithelial cells, after *H. pylori* infection, show higher levels of cytokines including IL-1ß, TNF-α and IL-8, a potent neutrophil-activating chemokine that apparently plays a central role in gastric diseases [1], [2]. *H. pylori* strains carrying the Cag-PAI (Cag Pathogenicity Island) induce a far stronger IL-8 response than Cag-negative strains, and this response depends on the activation of NF-kB (Nuclear Factor-kappaB) and the early-response transcription factor Activating Protein-1 [3]. IL-21 is constitutively expressed in gastric mucosa as well, and is more abundant in biopsy specimens from *H. pylori*-infected patients [4]. In response to pro-inflammatory stimuli by TNF-α and bacteria, gastric epithelial cells release several cytokines and induce the expression of NF-kB related genes, including metalloprotease-9 (MMP-9) [5], [6]. The integrity of the epithelial barrier is thus reduced, favouring pathogen infections. NF-kB is highly involved in the control of the transcription of the inflammatory mediators. The inducers of NF-kB activation such as TNF-α and IL-1ß produce elevated reactive oxygen species (ROS) levels suggesting that ROS act as common mediators of NF-kB activation [7] that could be blocked by antioxidants or overexpression of antioxidant enzymes superoxide dismutase (SOD) and catalase [8]. ROS including hydrogen peroxide (H_2_O_2_) are known to over-express IL-8 by activating oxidant-sensitive transcription factors such as NF-kB in gastric epithelial cells [9]. Patients with gastric ulcer have low levels of gastric antioxidants compared to normal mucosa [10]. Oxidative stress is also involved in the gastric damage induced by various agents including ethanol [11], [12]. Fruits and vegetables are suggested to confer protection for several pathologic conditions through their antioxidant effects.

*Rubus* berries, raspberries and blackberries, are considered to be a rich source of dietary antioxidants due to their high content of phenolic compounds. They contain high levels of ellagitannins (ETs) and ellagic acid conjugates (EAC), a class of polyphenols relatively uncommon in fruit and vegetables in our diet, being found only in few fruits, such as strawberries, pomegranates, muscadine grapes, some nuts, raspberries (*Rubus idaeus* L.) and blackberries (*Rubus fruticosus* L.) [13]. In *Rubus* berries, ETs and EAC are present in significant amounts (on average ca. 1.3 g/kg both in raspberries and blackberries, according to [14]) and represent the primary source of dietary ellagitannins [15].

An increasing number of evidences support for the health beneficial effects of plant polyphenols, including ETs. ETs received great attention mainly as beneficial nutrients against cardiovascular disease and cancer and show relevant anti-inflammatory and anti-angiogenic effects. Furthermore, ETs from pomegranate can modulate the intestinal inflammatory response.

While the biological activity of the pomegranate ETs, including the anti-inflammatory activity at the gastro-intestinal level [16] has been extensively studied, the ETs from *Rubus* berries, more frequently present in the Italian diet, are poorly investigated.

In this study, the attention is focused on ETs present in *Rubus* berries, raspberries and blackberries, to verify whether they are able to protect against gastric ulcer and how they interfere with the molecular cascade producing the inflammatory cell response at gastric level. At this aim, the efficacy of ETs was evaluated in a rat model of ethanol-induced gastric ulcer. *In vitro* ETs were assayed to investigate a) the inhibition of NF-kB translocation and driven transcription activity; b) the effect on IL-8 release in gastric epithelial cell line (AGS) stimulated with TNF-α, IL-1ß, H_2_O_2_, and ethanol.

## Materials and Methods

### Chemicals

All chemicals and solvents were of analytical ultra-pure grade. All the chromatographic solvents were HPLC grade or LC-MS grade for the MS experiments. Acetonitrile, acetone, methanol and diethyl ether were purchased from Sigma Aldrich (Milan, Italy). Hexane and formic acid were purchased from Carlo Erba (Milan, Italy). Ellagic acid standard (purity >98%) was purchased from Fluka (Steinheim, Germany). Sanguiin H-6 and lambertianin C were isolated as described in Gasperotti et al. 2010 [14]. Quercetin and polyethylene glycol 400 (PEG 400) were purchased from Sigma-Aldrich (Milan, Italy).

### Plant material and preparation of ETs enriched fraction

Blackberry (cv. Lochness), and raspberry (cv Tulameen) were grown in an experimental field in Vigalzano (Trento, Italy). The study involved only varieties widely disseminated for commercial purpose. No specific permissions were required for these locations, since the experimental field belongs to Fondazione Edmund Mach, San Michele all'Adige (TN, Italy). Berries were harvested at maturity and transported to the laboratory for the extraction. Before the extraction the samples were maintained at −20° C. 370 g of raspberries and 510 g of blackberries were extracted with a mixture acetone/water (70/30 v/v), as reported in Mattivi F. et al. [17], the ratio of fruit/solvent was 60 g/250 ml of solvent. Berries were homogenized with a 847-86 model Osterizer blender and centrifuged. The final volumes of the polyphenol-rich extracts were 1700 ml, respectively for blackberry, and 1240 ml for raspberry. Polyphenol-rich extracts were evaporated until dryness in a pear-shaped flask, using rotary evaporation under reduced pressure at 37 °C. The sample was diluted to 1 L with mixture methanol/water (30/70 v/v) and filtered using a Durapore 0.45 mm filter (Millipore, Vimodrone, Italy). The purification was carried out using an established method [14] with minor changes due to the high volume of the samples. Briefly, a column cartridge (10×4 cm), connected to a vacuum line, was packed with Sephadex LH-20 resin, pre-washed with 50 mL of methanol and then equilibrated with 100 ml of methanol/water (30/70 v/v). Fifty mL of the aqueous methanol extract was loaded and polyphenols, such as anthocyanins, were washed off with 500 ml of methanol/water (30/70 v/v). The fraction containing the ellagitannins was eluted using 350 mL of acetone/water (70/30 v/v). The ellagitannin-rich extracts were dried using rotary evaporation under reduced pressure at 37°C and reconstituted in 5 ml of methanol, added to 350 ml of diethyl ether and precipitated with hexane (700 ml). The ellagitannins-rich fraction was recovered by filtration and dried. The final weight of the precipitated ellagitannins enriched fraction was 480 mg for raspberry and 1485 mg for blackberry. An aliquot of the precipitate was further quantified by UPLC-PDA-MS to determine the amount of the main ellagitannins present. The quantification method applied was as reported in [14], and the ellagitannins were detected at 260 nm.

### Cell cultures and NF-κB assays

Human adenocarcinoma cells (AGS, CRL-1739, LGC Standard S.r.l., Milano, Italy) were grown at 37°C in DMEM F12 (Gibco-Invitrogen) supplemented with 100 units penicillin/ml, 100 mg streptomycin/ml, and 10% heat-inactivated foetal calf serum (Euroclone S.p.A, Pero, Italy), in a humidified atmosphere containing 5% CO_2_.

To evaluate the NF-κB driven transcription, cells were plated in 24-well plates (30000 cells/well); after 48 hours, cells were transfected by calcium-phosphate method with a plasmid containing the luciferase reporter gene under the control of NF-κB promoter, following a procedure previously reported [18]. After 16 hours, cells were placed in a medium deprived of FCS, and stimulated with TNF-α and IL-1β at 10 ng/ml. ETs were tested at 1–10 µg/ml; individual compounds at 0.5–10 µM. After 6 hours cells were harvested and luciferase assays were performed using Britelite™ Plus reagent (PerkinElmer Inc. Massachusetts, USA) according to manufacturer's instructions. Data were expressed considering 100% the luciferase activity related to the cytokine-induced NF-κB driven transcription.

Preliminary time-course experiments were performed to set the best conditions for further experiments. AGS were treated with TNF-α, IL-6, IL-21 and IL-8 and IL-1β 10 ng/ml, for 3, 6, 24, and 30 hrs. TNF-α and IL-1β only stimulated the NF-κB driven transcription, whereas the other cytokines were inactive. The maximal effect was observed at 6 hrs, and decreased at later times (Figure S1, a).

For the evaluation of the time-course of NF-κB (p65) translocation, AGS were treated with TNF-α and IL-1β 10 ng/ml, for 1,2,3, and 6 hrs. The maximal effect of nuclear translocation was observed at 1 hr and decreased at later times (Figure S1, b). These conditions were used for testing ETs (0.5–2 µg/ml) and individual compounds (0.5–10 µM). Parthenolide at 5 µM was used as reference inhibitor of NF-κB translocation. Results are the mean ± sd of three experiments in triplicate.

For the NF-κB (p65) nuclear translocation assay, AGS cells were plated at the concentration of 1.5 × 10^6^ cells/ml in 60-mm plates. After 48 hours, cells were treated for 1 hour with the inflammatory mediators and the extracts/compounds under study. Nuclear extracts were prepared using Nuclear Extraction Kit from Cayman Chemical Company (Michigan, USA) and stored at −80°C until assayed. The same quantity of total nuclear proteins, measured by the method of Bradford [19], was used to assess NF-κB nuclear translocation using the NF-κB (p65) transcription factor assay kit (Cayman) followed by spectroscopy (signal read 450 nm, 0.1 s). Data were expressed considering 100% the absorbance related to the cytokine-induced NF-κB nuclear translocation.

### Cell cultures and IL-8 release

Preliminary evaluation of IL-8 secretion was performed on AGS cells treated with TNF-α and IL-1β 10 ng/ml, for 1, 2, 3, and 6 hrs (Figure S1, c). Cells were grown in 24-well plates for 48 hrs (30000 cells/well) before the cytokine treatment. IL-8 was quantified by using Interleukin-8 High Sensitivity Human ELISA Set (Immunotools, Germany) using the method described below. Briefly, Corning 96 well EIA/RIA plates from Sigma-Aldrich (Milan, Italy), were coated with the antibody provided in the ELISA Set, overnight at 4°C. After blocking the reaction, 100 µl of samples in duplicate, were transferred into wells at room temperature for 1 hr. The amount of IL-8 in the samples was detected by spectroscopy (signal read 450 nm, 0.1 s) by the use of biotinylated and streptavidin-HRP conjugate antibodies, evaluating 3,5,3,5′-tetramethylbenzidine (TMB) substrate reaction. The quantification of IL-8 was done using an optimized standard curve supplied with the ELISA Set (1.0–240.0 pg/ml). The IL-8 secretion reached the maximum at 6 hrs and this time was selected for the experiments to test the effect of ETs (1–10 µg/mL) and individual compounds (0.25–5 µM), (Figure S1, c). Parthenolide (5 µM) was used as reference inhibitor of IL-8 secretion. Results are the mean ± sd of three experiments in triplicate.

### IL-8 secretion in AGS treated with H_2_O_2_ and ethanol

Cells were grown in 24-well plates for 48 hrs (30000 cells/well) and then incubated for 12 hrs in the presence of 500 µM H_2_O_2_, or for 24 hrs in the presence of 2% ethanol, following the procedure described in [9], with slight modifications. ETs were tested at 1–25 µg/mL. IL-8 was quantified as described above. Quercetin (10 µM) was used as reference inhibitor of IL-8 secretion. Results are the mean ± sd of three experiments in triplicate.

### Cytotoxicity assay

The integrity of the cell morphology before and after treatment was assessed by light microscope inspection. Cell viability of AGS was measured by MTT method [20]. No sign of cytotoxicity was observed in cells treated with ETs at 0.05–25 µg/mL. These concentrations are far below the content of ETs at gastric level after consumption of a portion of 100 g of berries at meals (100 mg for blackberry and 80 mg for raspberry). Ethanol and H_2_O_2_ were not toxic to AGS cells at the concentration used in the experiments.

### Animals

Thirty male Wistar rats (Charles River Laboratories, Calco, Lecco, Italy), weighing 175–200 g, were used. 3 Rats per cage were housed under constant environmental conditions (22 ± 1°C, 50 ± 5% relative humidity, 12-h light/12-h dark cycle), with free access to standard laboratory rat chow (014RF21C; Mucedola, Settimo Milanese, Milan, Italy) and tap water. Animals were acclimatized for a period of at least 7 days before the use. The study was approved (protocol number 16/2010) by the Animal Ethics Committee of University of Milan (Italy), and communicated to the Italian Ministry of Health, having regard to the article 7 of the D.L. 116/92. In addition, the study was carried out in strict accordance with the recommendations in the *Guide for the Care and Use of Laboratory Animals* published by the US National Institutes of Health (NIH Publication No. 85–23, revised 1996). All efforts were made to minimize animal suffering.

### Protocol

**Figure 1:**
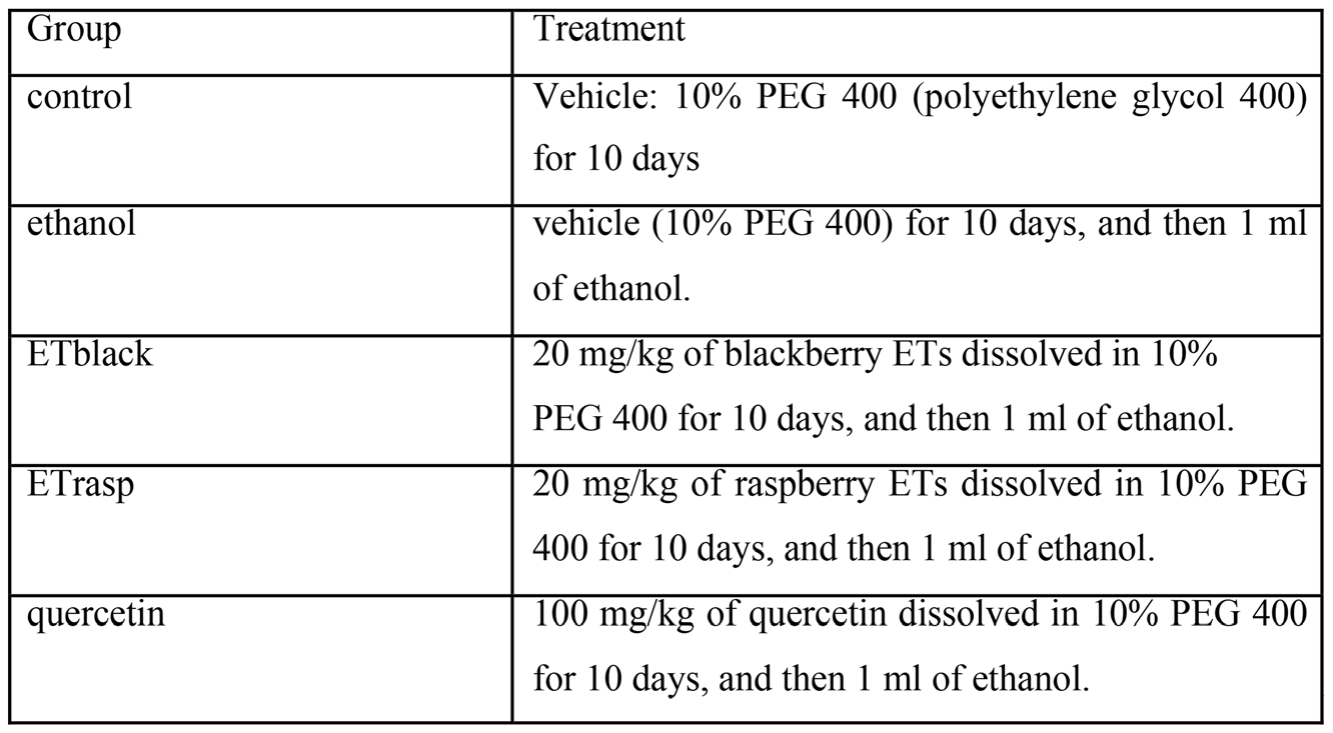
Experimental protocol for the in vivo study. Before the experiment, the animals were randomly divided in 5 groups (6 rats in each group) and treated intragastrically (i.g.) by gavage. The dose of ET enriched extracts was calculated on the basis of a daily consumption of 125 g of fresh fruit by a human healthy adult of 70 kg. The day before the induction of gastric lesions, rats were placed in individual metabolic cages and deprived of food, with free access to tap water for 20 h. The last administration of ETs extracts, quercetin or vehicle was given 120 min before ethanol treatment.

Before the experiment, the animals were randomly divided in 5 groups (6 rats in each group) and treated intragastrically (i.g.) by gavage, according to Figure 1.

The dose of ET enriched extracts was calculated on the basis of a daily consumption of 125 g of fresh fruit by a human healthy adult of 70 kg (Alvarez-Suarez 2011). The day before the induction of gastric lesions, rats were placed in individual metabolic cages and deprived of food, with free access to tap water for 20 h. The last administration of ETs extracts, quercetin (as positive control) or vehicle was given 120 min before ethanol treatment.

### Assessment of gastric mucosal damage

One hour after the administration of 1 ml of ethanol, rats were sacrificed under ether anesthesia by cervical dislocation; the stomach was removed and opened along the greater curvature. The stomach was rinsed with water, pinned open for microscopic examination by a microscope (Opmi 6; Carl Zeiss S.p.A., Arese, MI, Italy) and for photo-documentation by a digital camera (EOS 1100D, Canon Italia S.p.A., Cernusco Sul Naviglio, MI, Italy). Gastric hemorrhagic lesions in the glandular part were examined under a dissecting microscope (X10). Gastric damage was assessed in a blind manner. The Ulcer Index (UI) was obtained by a 0–3 scoring system based on the number and severity of the lesions [21], [22]. Severity was defined according to the length of the lesions: 0, no lesions; 1, lesions 1–2 mm; 2, lesions 2–3 mm; 3, lesions >3 mm. UI was calculated as the total number of lesions multiplied by their respective severity score.

### Preparation of gastric mucosa homogenates

Samples of 50 mg from normal and ulcerated rat gastric mucosa were homogenized in buffer A [10 mM TRIS-HCl (pH 8), 150 mM NaCl, 1 mM EDTA, 1 mM phenylmethylsulfonyl fluoride (PMSF), 2 µg/ml aprotinin, 2 µg/ml leupeptin, and 1% Triton X-100] using Tissue Lyser II (Qiagen) for 2 minutes at the highest frequency 30/s. The homogenates were centrifuged at 12,000 g for 10 min at 4° C and the supernatants collected, and stored at −80°C until use. Protein concentration was determined using Bradford protein assay (Bio-Rad) with bovine serum albumin as a standard.

### Cinc-1 (rat IL-8) release from gastric mucosa

The quantity of 40 µg of total proteins was used to assess Cinc-1 release using GRO/CINC-1 (rat) EIA kit (Enzo Life Sciences International, Inc., Plymouth Meeting, PA, USA). This kit uses a polyclonal antibody to rat GRO/CINC-1 labelled with the enzyme horseradish peroxidase. After a short incubation (10 minutes) the enzyme reaction was followed by spectroscopy (signal read 450 nm, 0.1 s). The concentration of rat GRO/CINC-1 in the samples was determined by interpolation with a GRO/CINC-1 standard curve. The results are expressed as pg of CINC-1 per mL of sample.

### Measurement of oxidative stress in rat gastric mucosa

The antioxidant capacity of the gastric mucosa homogenates was assessed by *Oxygen Radical Absorbance Capacity* (ORAC) assay. This method measures the oxidative degradation of fluorescein (Sigma-Aldrich Spa, Milan, Italy), after the addition of the free radical generator AAPH (2,2′-azobis(2-methylpropanimidamide)-dihydrochloride) (Sigma-Aldrich S.p.a., Milan, Italy). The oxidation of fluorescein by free radicals, leads to a decrease in fluorescence, prevented by the presence of antioxidant compounds. All reagents were prepared in 75 mM phosphate buffer, pH 7.4 and Trolox (4–160 µM) was used as the reference compound. Samples from gastric mucosa were suitably diluted in the phosphate buffer. Each well of a 96-well microplate contained 120 µL of fluorescein (0.07 µM) and 20 µL of the samples (corresponding to 5 µg protein) in a final volume of 200 µL assay solution. After the addition of AAPH (60 µL, 12 mM), the plate was shaken automatically for 2 seconds and the fluorescence was measured at 37°C every 2 min for 60 min with emission and excitation wavelengths of 528 and 485 nm, respectively, by using a microplate fluorescence reader (Victor™ ×3 Perkin Elmer 2030; Perkin Elmer Waltham MA, USA). The ORAC values were calculated as area under the curve and expressed as micromole of Trolox equivalent (TE) per gram of gastric mucosa sample ( µmol TE/g of gastric mucosa sample).

### Evaluation of CAT activity in rat gastric mucosa

CAT activity in gastric mucosa homogenates was determined by Catalase Assay Kit (Cayman Chemical, Ann Arbor, MI, USA), which utilizes the peroxidative function of CAT for the determination of the enzyme activity. The method is based on the reaction of the enzyme with methanol in the presence of an optimal concentration of H_2_O_2_. The formaldehyde produced is measured colorimetrically with 4-amino-3-hydrazino-5-mercapto-1,2,4-triazole as the chromogen using a microplate reader (Microplate Reader iMarkTM, Bio-Rad Laboratories S.r.l., Segrate, Italy) at 540 nm absorbance. Before starting the reaction, each well of a 96 well microplate contained 100 µL of diluted assay buffer, 30 µL of methanol and 20 µL of diluted gastric mucosa homogenates (2.5 µg/well). The amount of formaldehyde was calculated by means of a calibration curve of formaldehyde standard. CAT activity is expressed as units (U) of CAT per mg of proteins. One unit of CAT is defined as the amount of enzyme that will cause the formation of 1.0 nmol of formaldehyde per minute at 25°C.

### Evaluation of SOD activity in rat gastric mucosa

SOD activity was measured by using a SOD activity kit (Enzo Life Sciences International, Inc., Plymouth Meeting, PA, USA). This colorimetric assay evaluates the ability of SOD to reduce the superoxide ion concentration generated from the conversion of xanthine and oxygen to uric acid and hydrogen peroxide by xanthine oxidase. SOD activity was determined from percent inhibition of the rate of WST-1-formazan formation, a coloured product that absorbs light at 450 nm. Each sample was loaded in a 96 well microplate to the final amount of 6.25 µg/well. Immediately after the addition of xanthine, the plate was transferred to a microtiter plate reader (Victor™ ×3 Perkin Elmer 2030; Perkin Elmer Waltham MA, USA) and absorbance was read at 450 nm every minute for 10 minutes at room temperature, under 10 second orbital shake before each reading. The amount of SOD in the samples was calculated by correlating the inhibition percentage of WST-1-formazan formation with the logarithm of the SOD units in a standard calibration curve. SOD activity is expressed as U/mg of proteins.

### Statistical analysis

All data are expressed as mean ± sd, with the exception of the *in vivo* experiments expressed as mean ± se. Differences between means were calculated using the unpaired *t* test or one-way analysis of variance (ANOVA) followed by Tukey's post-hoc test for multiple group comparisons. Statistical analysis was done using GraphPad Prism 5.00 software (GraphPad Software Inc., San Diego, CA, USA); *p*<0.05 was considered statistically significant. IC_50_ was calculated using GraphPad Prism 5.00.

## Results

### ETs analysis and composition

**Figure 2:**
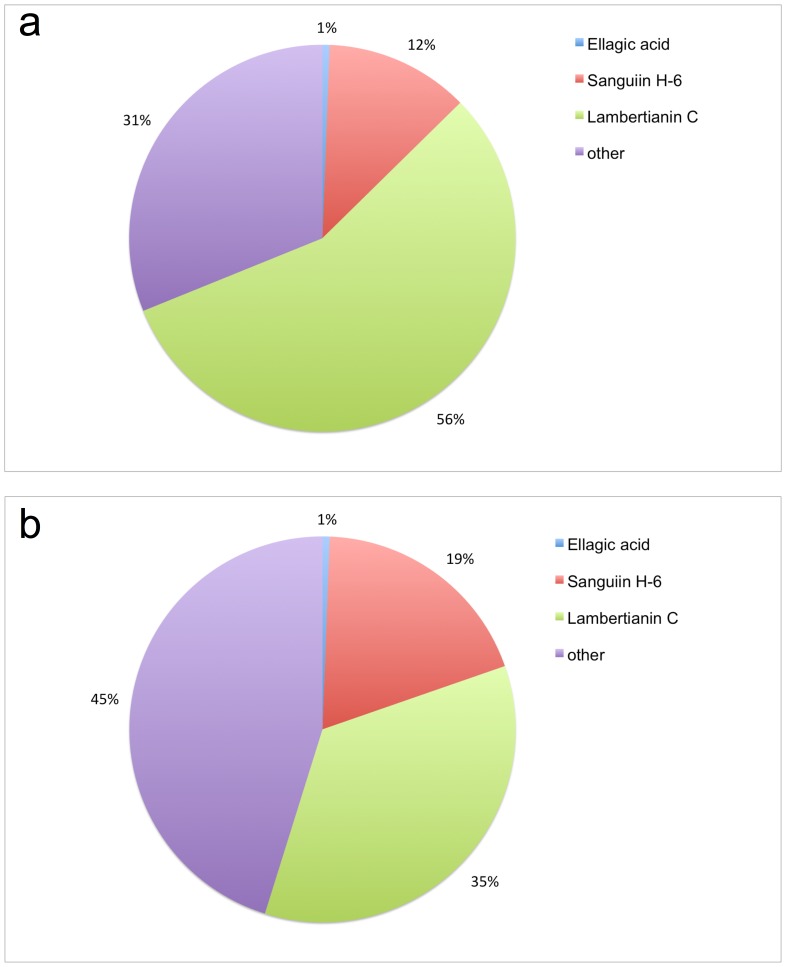
Composition of blackberry (ETblack) and raspberry (ETrasp) ellagitannins. Berries ETs were extracted with acetone/water 70:30, isolated by Sephadex LH 20 column chromatography, precipitated with hexane and quantified by UPLC-PDA-MS. Ellagitannins were detected at 260 nm.

Blackberry and raspberry, both members of genus *Rubus* are known to own similar profile of ellagitannins having sanguiin H-6 and lambertianin C as the main ellagitannins [14], [23]. In enriched fractions, ETs from blackberry (ETblack) corresponded to 343 mg/100 g of fresh fruits, ETs from raspberry (ETrasp) were 155 mg/100 g of fresh fruits. The composition of ETs was as follows: in ETblack sanguiin H-6 represented 12%, lambertianin C 56%, and ellagic acid 1% of the precipitate, while in ETrasp sanguiin H-6 represented 19%, lambertianin C 35%, and ellagic acid 1% (Figure 2).

### Effect of ETs on NF-κB driven transcription in AGS cells

**Figure 3:**
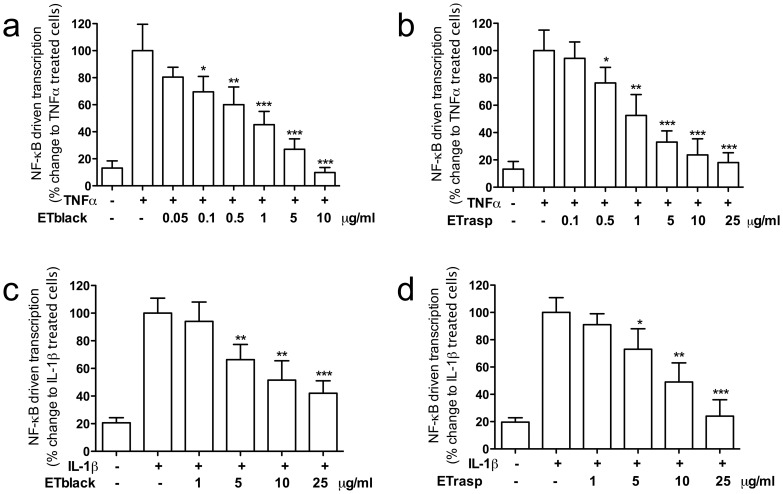
Effect of ETs from blackberry and raspberry on NF-κB driven transcription induced by TNFα and IL-1β. AGS cells at confluence were placed in a medium deprived of FCS, and stimulated with 10 ng/ml TNF-α (panel a and b) or (panel c and d) for 6 hrs. ETs were tested at 1–10 µg/ml; individual compounds at 0.5–10 µM. After 6 hours cells were harvested and luciferase assays were performed using Britelite™ Plus reagent (PerkinElmer Inc. Massachusetts, USA) according to manufacturer's instructions. Inhibition by 5 µM parthenolide, used as reference inhibitor, was 72% on TNF-α induced NF-κB driven transcription, and 71% on IL-1β induced NF-κB driven transcription. Results are the mean ± sd of three experiments in triplicate. * p<0.05, ** p<0.01, ***p<0.001.

Results are shown in Figure 3. ETblack and ETrasp inhibited the increase of NF-κB driven transcription induced by TNFα in a concentration dependent manner. IC_50_s were 0.67±0.17 and 1.7±0.6 µg/ml respectively for blackberry and raspberry. When IL-1β stimulated NF-κB driven transcription, inhibition by ETs was lower and comparable for blackberry and raspberry (IC_50_ 10.3±1.0 and 9.9±0.9, respectively for ETblack and ETrasp).

Lambertianin C and sanguiin H-6 inhibited NF-κB driven transcription stimulated by TNFα with IC_50_ of 2.7±0.45 and 1.5±0.35 µM, respectively. When NF-κB driven transcription was stimulated by IL-1β, the concentration required for obtaining 50% inhibition was 4.8±0.35 µM and 2.7±0.30 µM respectively for lambertianin C and sanguiin H-6 (Table 1).

**Table 1:** Effect of individual compounds sanguiin H-6 and lambertianin C on NF-κB pathway in human gastric epithelial AGS cells.

COMPOUND	NF-κB driven transcription IC_50_ µM (mean ± s.d.)		NF-κB translocation IC_50_ µM (mean ± s.d.)		IL-8 release IC_50_ µM (mean ± s.d.)	
	TNFα	IL-1β	TNFα	IL-1β	TNFα	IL-1β
Sanguiin H-6	1.5±0.35	2.7±0.30	0.87±0.16	1.9±0.23	0.58±0.05	1.0±0.06
Lambertianin C	2.7±0.45	4.8±0.35	1.2±0.39	1.7±0.30	0.57±0.03	1.2±0.06

### Effect of ETs on NF-κB (p65) translocation in AGS cells

**Figure 4:**
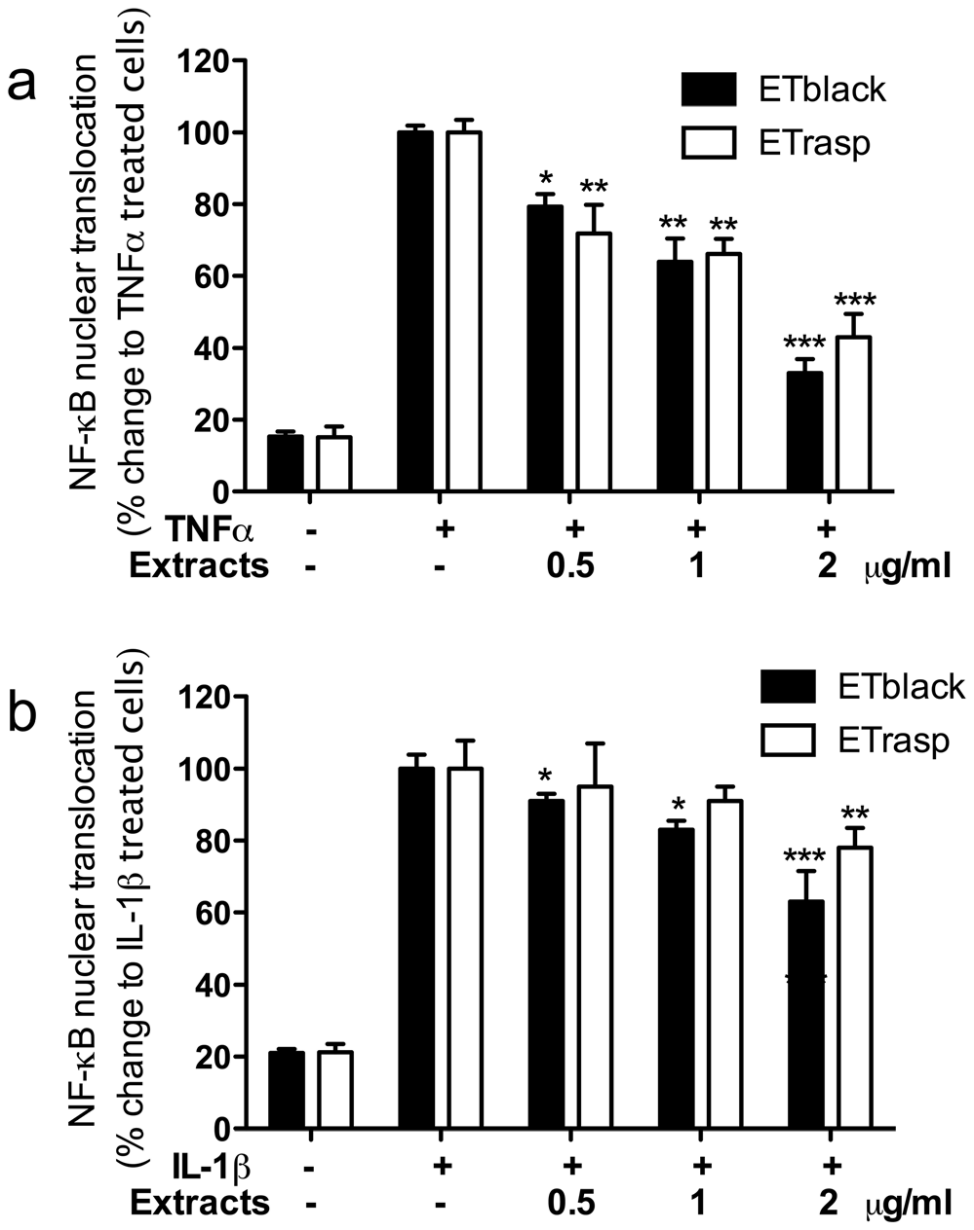
Effect of ETs from blackberry and raspberry on NF-κB nuclear translocation induced by TNFα and IL-1β. AGS cells at confluence were placed in a medium deprived of FCS, and stimulated with 10 ng/ml TNF-α (panel a) or IL-1β (panel b) for 1 hr. AGS cells were plated at the concentration of 1.5 × 10^6^ cells/ml in 60-mm plates. NF-κB nuclear translocation was assessed using the NF-κB (p65) transcription factor assay kit (Cayman) followed by spectroscopy (signal read 450 nm, 0.1 s). Inhibition by 5 µM parthenolide used as reference inhibitor was 37% on TNF-α induced NF-κB nuclear translocation, and 40% on IL-1β induced nuclear translocation. Results are the mean ± sd of three experiments in triplicate. * p<0.05, ** p<0.01, ***p<0.001.

ETblack and ETrasp, at 2 µg/ml, inhibited TNFα-induced translocation by 67% and 57% respectively (Figure 4, panel a). The inhibitory effect of ETs on IL-1β induced translocation was much lower (37% and 22% at 2 µg/ml of ETblack and ETrasp respectively) (Figure 4, panel b).

Lambertianin C at 5 µM and sanguiin H-6 at 2.5 µM reduced translocation of NF-κB at the basal levels (control without stimulus). IC_50_s were 1.2±0.39 and 0.87±0.16 µM for lambertianin C and sanguiin H-6, respectively. When cells were treated with IL-1β, IC_50_s were 1.7±0.30 and 1.9±0.23 µM for lambertianin C and sanguiin H-6, respectively (Table 1).

### Effect of ETs on IL-8 release in AGS cells

**Figure 5:**
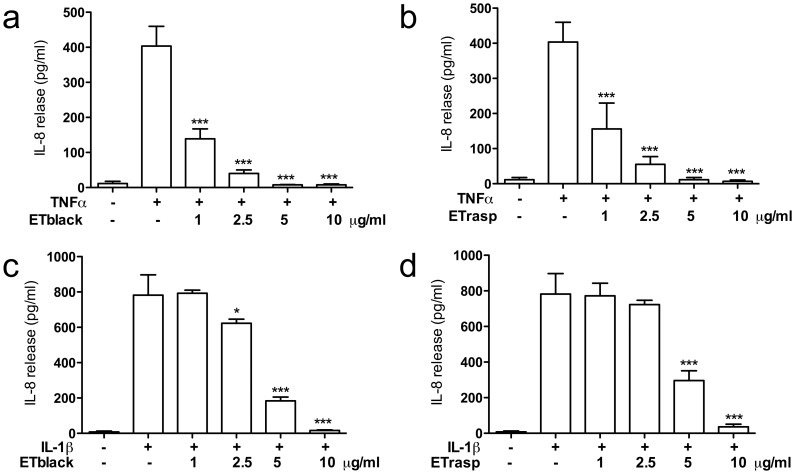
Effect of ETs from blackberry and raspberry on IL-8 release induced by TNFα and IL-1β. AGS cells at confluence were placed in a medium deprived of FCS, and stimulated with 10 ng/ml TNF-α (panel a and b) or IL-1β (panel c and d) for 6 hrs. IL-8 was quantified by using Interleukin-8 High Sensitivity Human ELISA Set (Immunotools, Germany). The amount of IL-8 in the samples was detected by spectroscopy (signal read 450 nm, 0.1 s) by the use of biotinylated and streptavidin-HRP conjugate antibodies, evaluating 3,5,3,5′-tetramethylbenzidine (TMB) substrate reaction. Inhibition by 5 µM parthenolide, used as reference inhibitor, was 70% for both TNF-α and IL-1β induced IL-8 secretion. Results are the mean ± sd of three experiments in triplicate. * p<0.05, ***p<0.001.

Since IL-8 is widely involved in gastric inflammation, and its expression is NF-κB dependent, the following experiments were devoted to evaluate the effect of the extracts/individual compounds on IL-8 secretion induced by TNFα and IL-1β in AGS. ETblack and ETrasp inhibited the increase of IL-8 release induced by TNFα in a concentration dependent manner (Figure 5a, 5b). IC_50_s were 0.69±0.06 and 0.77±0.07 µg/ml respectively for ETblack and ETrasp. When IL-8 release was stimulated with IL-1β, ETs inhibition was lower and IC_50_s were 4.0±0.74 and 4.5±0.08 µg/ml, respectively for ETblack and ETrasp (Figure 5c, 5d).

Lambertianin C and sanguiin H-6, at 2.5 µM, totally abolished the release of IL-8 stimulated by TNFα and by IL-1β. IC_50_s were 0.57±0.03 and 0.58±0.05 µM for lambertianin C and sanguiin H-6, respectively in cells stimulated with TNFα; when cells were stimulated by IL-1β IC_50_s were 1.2±0.06 and 1.03±0.06 µM for lambertianin C and sanguiin H-6, respectively (Table 1).

**Figure 6:**
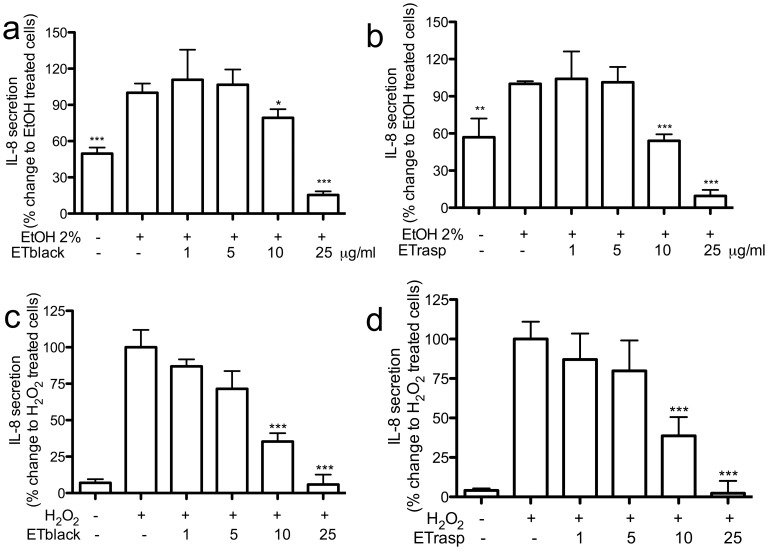
Effect of ETs from blackberry and raspberry on IL-8 release induced by EtOH and H_2_O_2_. Cells were grown in 24-well plates for 48 hrs (30000 cells/well) and then incubated for 12 hrs in the presence of 500 µM H_2_O_2_ (panels a and b), or for 24 hrs in the presence of 2% ethanol (panels c and d). IL-8 was quantified by using Interleukin-8 High Sensitivity Human ELISA Set (Immunotools, Germany). The amount of IL-8 in the samples was detected by spectroscopy (signal read 450 nm, 0.1 s) by the use of biotinylated and streptavidin-HRP conjugate antibodies, evaluating 3,5,3,5′-tetramethylbenzidine (TMB) substrate reaction. 10 µM quercetin, used as reference inhibitor, completely inhibited EtOH and H_2_O_2_ induced IL-8 secretion. Results are the mean ± sd of three experiments in triplicate. * p<0.05, ** p<0.01, ***p<0.001.

In cells treated with ethanol the secretion of IL-8 was twice higher than in control cells. The addition of ETs reduced the release of IL-8 and the IC_50_s were 11.5 ± 0.9 and 9.8 ± 0.1 µg/mL, respectively for ETblack and ETrasp (Figure 6a, 6b). When cells were stimulated by H_2_O_2_, IL-8 secretion was ten times higher than in control cells and the effect was inhibited by ETs (IC_50_s were 7.0 ± 2.1 and 8.2 ± 1.5 µg/mL, respectively for ETblack and ETrasp, Figure 6c, 6d).

### Protective effect of ETs on gastric injury in rats

No difference in weight gain was observed in control and treated animals (Table S1).

**Figure 7:**
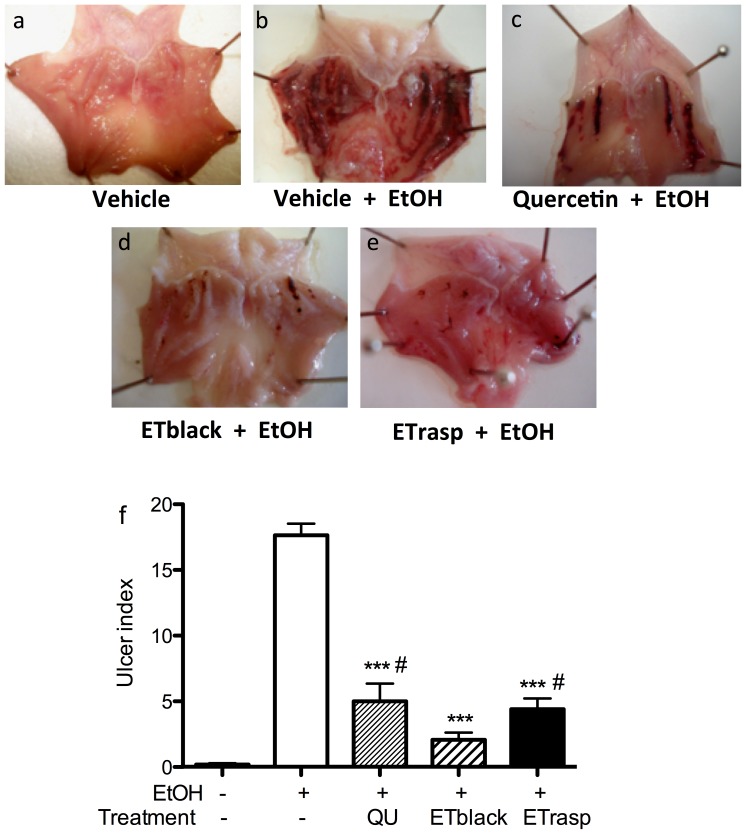
Protective effect of ETs from blackberry and raspberry against ethanol induced gastric injury. Wistar rats were randomly divided in 5 groups (6 rats in each group). Controls were treated daily with vehicle (10% polyethylene glycol 400; PEG 400) for 10 days. Ethanol group received the vehicle (10% PEG 400) daily for 10 days, and then 1 ml of ethanol. ETblack group received 20 mg/kg of blackberry ETs dissolved in 10% PEG 400 for 10 days, and then 1 ml of ethanol. ETrasp group recived 20 mg/kg of raspberry ETs dissolved in 10% PEG 400 for 10 days, and then 1 ml of ethanol. Quercetin group (positive control) received 100 mg/kg of quercetin dissolved in 10% PEG 400 for 10 days, and then 1 ml of ethanol. The last administration of ETs, quercetin or vehicle was given 120 min before ethanol. Treatment was performed intragastrically by gavage. Gastric damage was assessed in a blind manner by a scoring system based on the number and severity of the lesions: 0, no lesions; 1, lesions 1–2 mm; 2, lesions 2–3 mm; 3, lesions >3 mm. Ulcer Index was calculated as the total number of lesions multiplied by their respective severity score. Results are the mean ± se, n = 6. ***p<0.001.

Gastric lesions (UI score 17.8±0.8; *p*<0.001 as compared to controls animals) were found in the mucosa of ethanol treated rats consisting of elongated bands usually parallel to the long axis of the stomach. Ulcers were located mostly in the corpus, the portion of stomach secreting acid and pepsin. No visible lesions developed in the non-secretory part of the stomach (Figure 7a-e). ETblack and ETrasp showed a high protective effect against ethanol injury, when given once a day for 10 consecutive days before the administration of 1 ml ethanol. Both extracts significantly reduced gastric lesions by 88% and 75%, respectively ((*p*<0.001, Figure 7d, 7e, 7f). Quercetin (100 mg/kg day) used as positive control, reduced UI by 70%. In addition, a significant difference (*p*<0.05) was found between the effect of ETblack and ETrasp and between ETblack and quercetin (Figure 7c, 7f).

### Effect of ETs on biochemical parameters ex vivo

**Figure 8:**
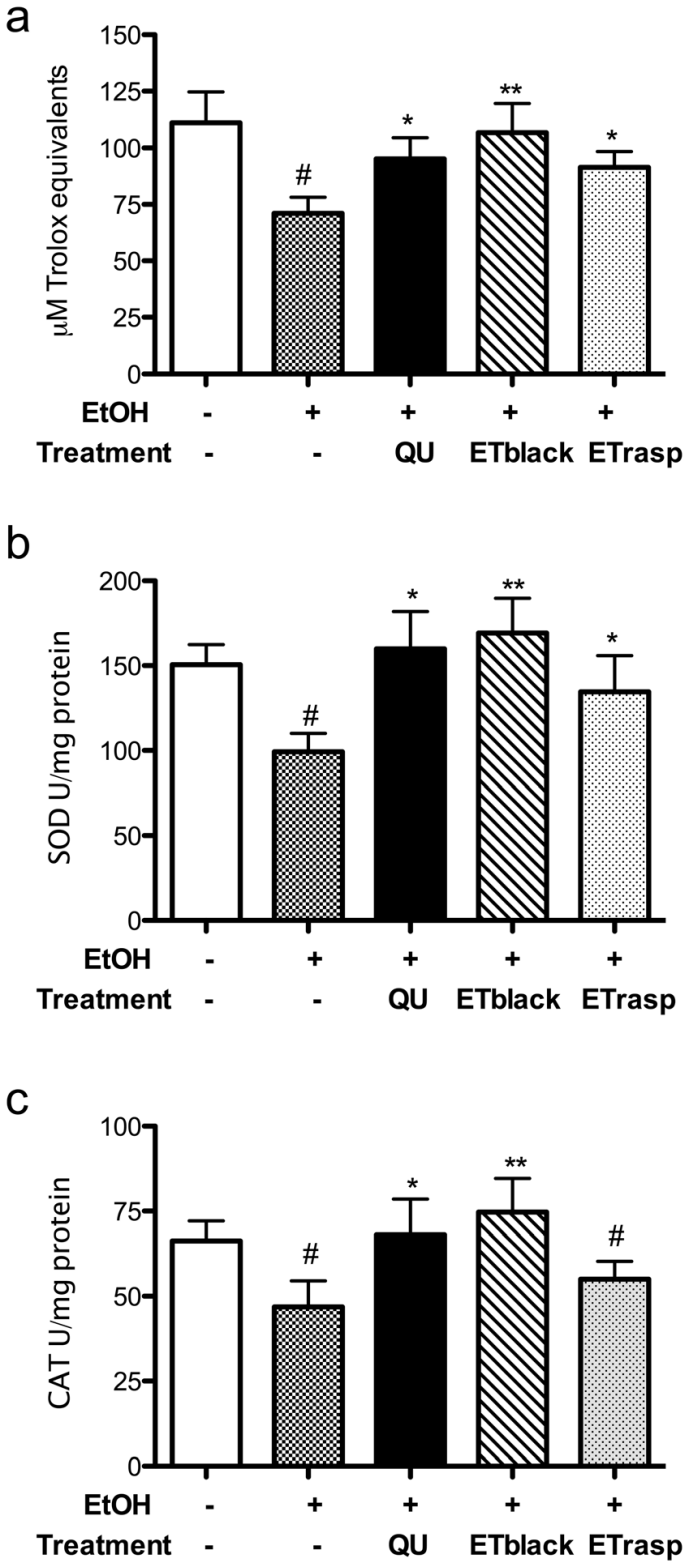
Effect of ETs from blackberry and raspberry on biochemical parameters *ex vivo*. Rat gastric mucosa was homogenized in buffer A [10 mM TRIS-HCl (pH 8), 150 mM NaCl, 1 mM EDTA, 1 mM phenylmethylsulfonyl fluoride (PMSF), 2 µg/ml aprotinin, 2 µg/ml leupeptin, and 1% Triton X-100] using Tissue Lyser II (Qiagen) for 2 minutes at the highest frequency 30/s. The homogenates were centrifuged at 12,000 g for 10 min at 4° C. The supernatants were collected and stored at −80°C until use. The antioxidant capacity of the gastric mucosa homogenates was assessed by *Oxygen Radical Absorbance Capacity* (ORAC) assay and Trolox (4–160 µM) was used as the reference compound. The ORAC values were calculated as area under the curve and expressed as micromole of Trolox equivalent (TE) per gram of gastric mucosa sample ( µmol TE/g of gastric mucosa sample). SOD activity was determined from percent inhibition of the rate of WST-1-formazan formation, a coloured product that absorbs light at 450 nm. The amount of SOD in the samples was calculated by correlating the inhibition percentage of WST-1-formazan formation with the logarithm of the SOD units in a standard calibration curve. SOD activity is expressed as U/mg of proteins. CAT activity was determined by Catalase Assay Kit, a method based on the reaction of the enzyme with methanol in the presence of an optimal concentration of H_2_O_2_. The formaldehyde produced is measured colorimetrically at 540 nm with 4-amino-3-hydrazino-5-mercapto-1,2,4-triazole as the chromogen using a microplate reader. CAT activity is expressed as units (U) of CAT per mg of proteins. One unit of CAT is defined as the amount of enzyme that causes the formation of 1.0 nmol of formaldehyde per minute at 25°C. Results are the mean ± se, n = 6. * p<0.05, ** p<0.01.

In the gastric mucosa of rats treated with ethanol, the biochemical parameters relative to the oxidative state were modified with respect to control animals: Trolox equivalents, SOD and CAT activities were all reduced, indicating an unbalance of the steady state of the tissue versus an oxidant condition (Figure 8). Both ETblack and ETrasp counteracted the oxidant effect of ethanol, ETblack being more efficient than ETrasp. In rats treated with ETblack, Trolox equivalents, SOD and CAT levels returned to the values found in control animals.

**Figure 9:**
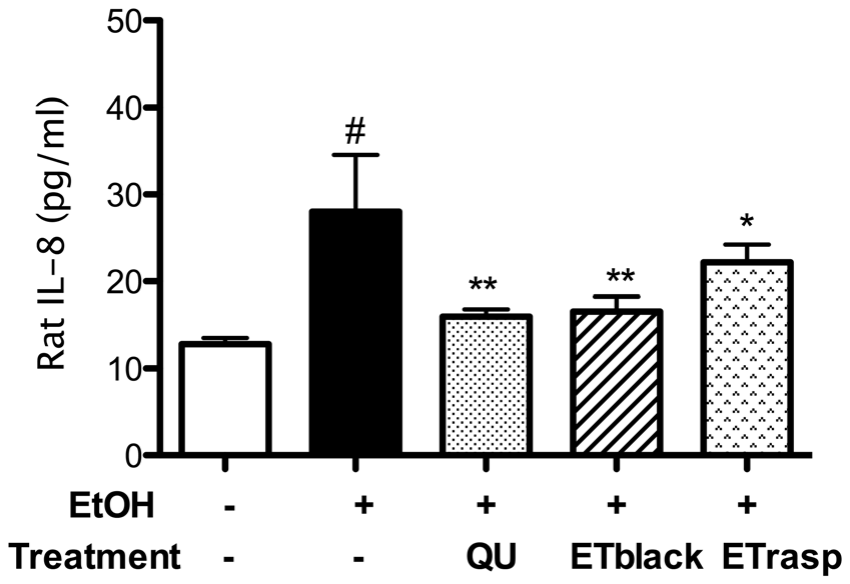
Effect of ETs from blackberry and raspberry on CINC-1 (IL-8 homologue) *ex vivo*. CINC-1 release was evaluated using GRO/CINC-1 (rat) EIA kit which uses a polyclonal antibody to rat GRO/CINC-1 labelled with the enzyme horseradish peroxidase. After a short incubation (10 minutes) the enzyme reaction was followed by spectroscopy (signal read 450 nm, 0.1 s). The concentration of rat GRO/CINC-1 in the samples was determined by interpolation with a GRO/CINC-1 standard curve. The results (mean ± se, n = 6) are expressed as pg of CINC-1 per mL of sample. * p<0.05, ** p<0.01.

The administration of ethanol caused a higher release of CINC-1 (the rat homologue of human IL-8) from 12.8 pg/ml in the tissue from control animals to 28 pg/ml in ethanol treated rats (Figure 9). In animals treated with ETblack and ETrasp the amount of CINC-1 was significantly lower with respect to ethanol (16.5 ± 1.9 and 22.2 ± 2.3 pg/ml, respectively). In rats treated with quercetin as positive control CINC-1 levels were 15.9 ± 0.72 pg/ml. The effect of ETs on CINC-1 was associated to a decrease of NF-κB translocation in ETblack and ETrasp animals in comparison with control and ethanol treated animals. In the tissue of ETblack and ETrasp animals, NF-κB translocation was inhibited by 38±0.11% (mean ± sd, n = 6, p<0.001) and 72±1.6% (n = 6, p<0.0001) respectively, with respect to ethanol. No difference was observed between control and ethanol group. No data are present in the literature as regards NF-κB nuclear translocation in vivo in the animal model of ethanol-induced ulcer. An explanation is that the damage of the tissue does not allow to properly isolate the nuclear fraction.

## Discussion

Polyphenols are a class of heterogeneous compounds that are recognized to possess several biological activities. Among polyphenols, flavonoids, anthocyanins, and to a lesser extent proanthocyanidins, were the most studied, while the biological effects of ETs were randomly investigated. ETs are contained in medicinal plants [24] and in some fruits such as pomegranate, blackcurrant, nuts, and muscadine grapes [13]. The majority of studies (many *in vitro* and few in humans), devoted to investigate the beneficial effects of dietary ETs, focused on cardiovascular diseases and cancer [23], [25], [26]. Almost all studies were conducted with ETs enriched extracts derived from pomegranate [27], while ETs from other sources such as *Rubus*, frequently present in the European diet and highly appreciated for their flavour, are poorly investigated [28]. The chemical composition of the ETs fraction is depending of the fruit source, and sanguiin H-6 and lambertianin C represent the main compounds in blackberry and raspberry [14].

Our interest for the ETs and peptic ulcer derives from the observation that plant rich in tannins have a traditional use for treating ulcer and hydrolysable tannins show anti-bacterial activity against *Helicobacter pylori* [24], [29]. At gastric level, the acidic conditions and the gastric enzymes are unable to hydrolize the original ellagitannin [30]. Metabolism of ETs takes place in the intestine where the physiological pH of the small intestine causes the hydrolysis of ETs and the release of ellagic acid. The latter is then metabolized by the gut microflora to several metabolites called urolithins [30]. Both ellagic acid and urolithins are then absorbed and found in plasma [15]. Therefore the effects observed at the gastric level are totally associated to the unmodified ellagitannin structure, and are not related to the metabolic transformation in contrast to that occurring in the intestine or other systemic situations.

This study reports for the first time that ETs from blackberries and raspberries are able to protect the stomach against the gastric lesions caused by ethanol. It is remarkable that the effect was obtained at a dose of ETs comparable with the amount consumed in a portion of berries of 125 g, corresponding to the weight of the content of the baskets distributed on the market. The anti-ulcer effect of blackberry, measured as UI, was higher than that of raspberry. Blackberry extract contained a higher amount of ETs (343 mg/100 g of fresh fruits) than raspberry (155 mg/100 g of fresh fruit); then, the difference in ETs content could account for the different response. In a previous study Alvarez-Suarez et al. also found that strawberry extract inhibited the onset of gastric ulcer in a rat model of ethanol-induced gastritis [12]; however, their study was conducted with extracts enriched with the anthocyanin fraction of the fruits and linked the gastro-protective effect to the antioxidant properties of anthocyanin mixture. Our results indicate that also ETs can contribute to the anti-ulcer beneficial effects of berries.

The present study show that the low levels of Trolox equivalents, SOD and CAT in the gastric mucosa of ethanol group returned at normal values in the mucosa of animals treated with ETs. In addition, the reduction of the severity of the lesions in ETs treated rats with respect to ethanol alone was also associated to a concomitant reduction of the release of CINC-1, the rat homologue of human IL-8. Since the IL-8 expression and secretion in gastric epithelial cells are mainly regulated by redox-sensitive transcription of NF-κB [9], it was assumed that the suppression of NF-κB mediated cell signalling could be a mechanism for inhibition of the inflammatory process by ETs. This assumption was confirmed by the reduction of NF-κB nuclear translocation *ex vivo* observed in animals treated with ETs with respect to ethanol. According to our *in vivo* experiments then, the action of ETs is not limited to their antioxidant properties but also to other molecular mechanisms involving, at least in part, NF-κB pathway.

*In vitro* experiments with AGS gastric epithelial cells confirm and further characterize the molecular function of ETs. The activity of ETs was tested in cells exposed to various inflammatory challengers known to activate NF-κB cell signalling: cytokines TNF-α and IL-1β, or the pro-oxidant agents ethanol and H_2_O_2_. The results show that ETs interfere with the metabolic cascade deriving from the activation and translocation of NF-κB that in turn activates the transcription of targeted genes including that of IL-8. ETs inhibited IL-8 secretion also in cells stimulated by pro-oxidant agents such as ethanol and H_2_O_2_ at concentrations slightly higher (7–8 µg/mL) than those required when cells were stimulated by TNF-α or IL-1β cytokines. The results of these experiments mimic those observed in the animal model of ethanol-induced gastric inflammation.

In AGS stimulated by cytokines (TNF-α and IL-1β) ETs inhibited the translocation and driven-transcription of NF-κB and the release of IL-8. The concentration necessary to obtain 50% inhibition (IC_50_) ranged between 1 and 2 µg/mL of extracts. Considering a volume of rat gastric juice of 20 ml, the consumption of 125 g of fresh fruits by an adult allows to reach a concentration of ETs in the stomach around 6.4 mg/ml, which is 10^3^ higher than the concentration required to inhibit translocation of NF-κB in the nucleus and then activation of gene transcription. The effect by ETs was less potent in cells stimulated by IL-1β compared to cells stimulated by TNF-α. The reasons for this observation is presently unknown, but it was previously shown that antioxidants showed more potent inhibitory effect on TNF-α-induced IL-8 secretion compared with IL-1β-induced IL-8 secretion [31]. An explanation may reside in the different role of ROS in the NF-κB activation by TNF-α and IL-1β [32], [33] and how/when ETs block ROS generation. At this stage of the research explanations can be only speculative. The cascade of cell events generated by TNF-α for the activation of NF-κB is different as compared to IL-1β [34], and ETs could interact at different sites along the cascade. Either ETs can function as binding inhibitors at the receptor sites of TNF-α (TNFR1) and IL-1β (IL-1R1). The compounds may act at different levels in the complex regulatory process of NF-κB pathway, a question that can be a matter for future studies.

ETs used in this study are a mixture of compounds that contribute to the total activity of the extract. ETblack and ETrasp have qualitatively similar profile both having lambertianin C and sanguiin H-6 as the main ellagitannin, but different composition: the compounds represent 47% and 74% of the total ETs in ETrasp and ETblack, respectively. The experiments with single ET were performed to study the reciprocal contribution of each. Both showed an impressive inhibition of NF-κB activation and IL-8 secretion and were equally potent; then, the contribution to the overall effect of ETs mixture depends on their amount in the extract. As found in all the experiments, ETblack containing higher amounts possess higher activity than ETrasp. Since the *in vitro* studies performed with the mixtures are well reproducible in the *in vivo* animal model, it is conceivable that lambertianin C and sanguiin H-6 are the active principles of ETblack and ETrasp.

In conclusion we have shown that ETs from *Rubus* berries efficiently protect against the onset of gastric ulcer in a rat animal model. ETs act through the inhibition of the NF-κB cascade either directly on the cell response to pro-inflammatory cytokines or as antioxidant agents inhibiting ROS generated in several inflammatory conditions including ethanol damage or *Helicobacter pylori* infection. For these reasons, the study of the biological activity of this class of compounds deserves more attention. Further studies will consider experimental models of *Helicobacter pylori* dependent ulcer and the immunomodulatory effect of these compounds in the context of peptic ulcer. Polyphenols such as ETs and anthocyanins are constituents of the fruits of *Rubus spp*. It would be of interest to study if and how these two classes of important dietary components interact at gastric level for the modulation of inflammatory response.

The outcome of this research will allow to draw the attention of the clinical/dietology community towards the benefits of the fruits of *Rubus* spp. as integration in dietary regimens designed for inflammatory gastrointestinal diseases.

## Supporting Information

Figure S1 **Time course experiments in order to set the best conditions for further experiments with the compounds under study.** AGS were treated with TNF-α, IL-6, IL-21 and IL-8 and IL-1β 10 ng/ml, for 3, 6, 24, and 30 hrs. TNF-α and IL-1β only stimulated the NF-κB driven transcription, whereas the other cytokines were inactive. The maximal effect was observed at 6 hrs, and decreased at later times (panel a). For the evaluation of the time-course of NF-κB (p65) translocation, AGS were treated with TNF-α and IL-1β 10 ng/ml, for 1,2,3, and 6 hrs. The maximal effect of nuclear translocation was observed at 1 hr and decreased at later times (panel b). Preliminary evaluation of IL-8 secretion was performed on AGS cells treated with TNF-α and IL-1β 10 ng/ml, for 1, 2, 3, and 6 hrs. IL-8 secretion was higher at 6 hrs and this time was selected for ETs and for individual compounds evaluation (panel c).
(TIFF)

Table S1 **Effect of the treatment with the extracts on rat weight.** No difference in weight gain was observed in the 4 groups of rats (group 2–5), as compared with controls animals (group 1) receiving only the chronic administration of vehicle (PEG 400).
(DOCX)
