# Supplementary figures and images for: Ellagitannins from Rubus Berries for the Control of Gastric Inflammation: In Vitro and In Vivo Studies
Source: PLoS One. 2013 Aug 5;8(8):e71762. doi: 10.1371/journal.pone.0071762 (PMC3733869; doi:10.1371/journal.pone.0071762)

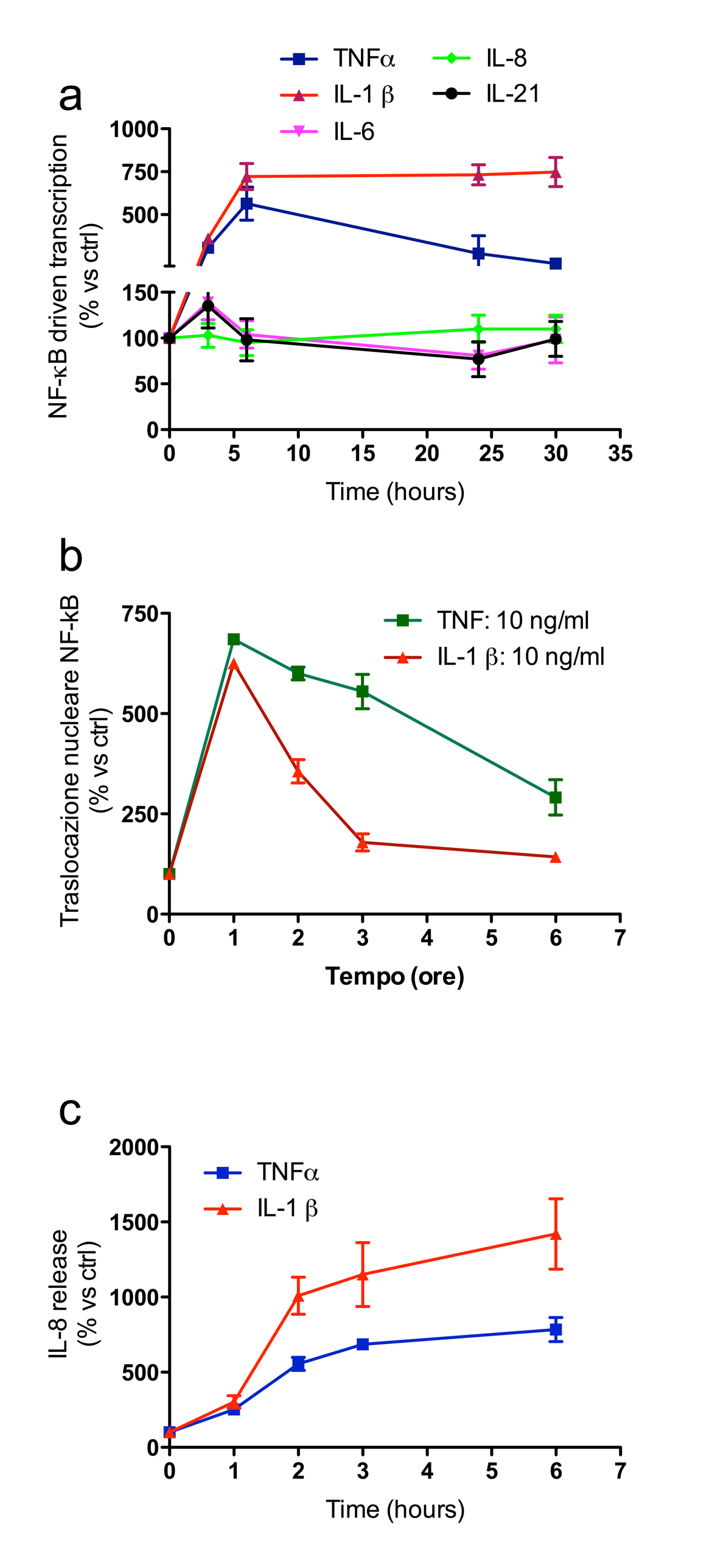

Supplement: Figure S1 — Time course experiments in order to set the best conditions for further experiments with the compounds under study. AGS were treated with TNF-α, IL-6, IL-21 and IL-8 and IL-1β 10 ng/ml, for 3, 6, 24, and 30 hrs. TNF-α and IL-1β only stimulated the NF-κB driven transcription, whereas the other cytokines were inactive. The maximal effect was observed at 6 hrs, and decreased at later times (panel a). For the evaluation of the time-course of NF-κB (p65) translocation, AGS were treated with TNF-α and IL-1β 10 ng/ml, for 1,2,3, and 6 hrs. The maximal effect of nuclear translocation was observed at 1 hr and decreased at later times (panel b). Preliminary evaluation of IL-8 secretion was performed on AGS cells treated with TNF-α and IL-1β 10 ng/ml, for 1, 2, 3, and 6 hrs. IL-8 secretion was higher at 6 hrs and this time was selected for ETs and for individual compounds evaluation (panel c). (TIFF) [file pone.0071762.s001.tiff]
