# Supplementary material for: Ellagitannins from Rubus Berries for the Control of Gastric Inflammation: In Vitro and In Vivo Studies
Source: PLoS One. 2013 Aug 5;8(8):e71762. doi: 10.1371/journal.pone.0071762 (PMC3733869; doi:10.1371/journal.pone.0071762)
Supplement: Table S1 — Effect of the treatment with the extracts on rat weight. No difference in weight gain was observed in the 4 groups of rats (group 2–5), as compared with controls animals (group 1) receiving only the chronic administration of vehicle (PEG 400). (DOCX) [file pone.0071762.s002.docx]

| Group | Initial weight  (g) | Final weight  (g) |
| --- | --- | --- |
| 1 | 185.3 ± 6.7 | 214.8 ± 4.9 |
| 2 | 191.4 ± 5.3 | 221.3 ± 7.9 |
| 3 | 180.6 ± 8,2 | 211.7 ± 6.5 |
| 4 | 183.0 ±7.4 | 215.3 ± 5.3 |
| 5 | 190.2 ± 5.8 | 222.5 ± 8.4 |
